# Divergent responses of leaf traits to latitudinal gradients in natural and planted forests

**DOI:** 10.48130/forres-0026-0002

**Published:** 2026-01-30

**Authors:** Jianxiao Su, Jiali Xu, Mengyao Yu, Jie Gao

**Affiliations:** 1 Key Laboratory for the Conservation and Regulation Biology of Species in Special Environments, College of life science, Xinjiang Normal University, Urumqi 830054, China; 2 Key Laboratory of Earth Surface Processes of Ministry of Education, College of Urban and Environmental Sciences, Peking University, Beijing 100871, China

**Keywords:** Leaf functional traits, Latitudinal gradient, Planted forest vs natural forest, Trait–environment coupling, Resource utilization strategy, Climate–soil–forest interactions

## Abstract

Leaf functional traits are key indicators of plant resource use and environmental adaptation, playing a crucial role in regulating carbon cycling and ecosystem stability. However, how leaf traits respond to latitudinal gradients in natural and planted forests remains insufficiently understood. Based on 482 forest plots across China (105 natural and 377 planted forests) surveyed from 2008 to 2020, latitudinal variation in specific leaf area (SLA), leaf dry matter content (LDMC), leaf nitrogen (LN), and leaf phosphorus (LP) were examined using quadratic polynomial fitting, variance partitioning, CatBoost analysis, and structural equation modeling (SEM). Natural and planted forests exhibited marked structural differences: natural forests had higher species richness and high stand diversity, whereas planted forests were structurally simplified, younger, and strongly shaped by management. Planted forests showed pronounced non-monotonic variation along latitude, with SLA and LP peaking at mid-latitudes, whereas natural forests exhibited weaker and more gradual latitudinal changes. Climatic and soil factors jointly dominated trait variation in natural forests, while latitude and stand structure were the primary determinants in planted forests. SEM further revealed that latitude affected leaf traits through indirect pathways mediated by climate, soil, and stand factors, with opposite effects between forest types. Natural forests showed consistent and climate-dominated trait responses, with soil properties mediating these effects in predictable ways, reflecting long-term environmental filtering. In contrast, planted forests exhibited greater short-term environmental plasticity. These findings highlight divergent mechanisms of trait–environment relationships between natural and planted forests and underscore the importance of integrating stand structure and climate matching in planted forests management to enhance ecological resilience and carbon sequestration under global change.

## Introduction

Plants adopt a series of coordinated strategies to acquire, use, and conserve resources in response to environmental constraints, and these strategies are strongly mediated by their functional traits^[1,2]^. Leaf functional traits—such as specific leaf area (SLA), leaf dry matter content (LDMC), leaf nitrogen (LN), and leaf phosphorus (LP)—serve as key indicators of these strategies by reflecting the balance between rapid resource acquisition and conservative resource retention^[3,4]^. For instance, high SLA and LN values are typically associated with acquisitive strategies characterized by fast photosynthetic turnover and high growth rates, whereas high LDMC and low SLA represent conservative strategies that enhance tissue durability and resource-use efficiency under stressful conditions^[5]^. These trait combinations are not only essential for determining species-level performance but also scale up to influence community assembly and ecosystem functioning by shaping patterns of primary productivity, carbon sequestration, and nutrient cycling^[6]^. Consequently, understanding variation in leaf functional traits provides crucial insights into the mechanisms linking plant resource-use strategies with ecosystem processes and stability.

Latitudinal gradients offer a powerful natural experiment for examining how plants adjust their functional traits to broad-scale environmental variation^[7]^. Latitude integrates multiple climatic drivers—such as mean annual temperature, precipitation regimes, radiation, and season length—that are known to shape plant metabolic rates, nutrient-use strategies, and life-history trade-offs^[8]^. As plants experience increasingly colder temperatures, shorter growing seasons, and reduced nutrient availability toward higher latitudes, they typically shift from acquisitive to more conservative resource-use strategies, reflected in systematic changes in leaf traits such as SLA, LDMC, LN, and LP^[9]^. However, these responses are often curvilinear rather than strictly monotonic, frequently displaying quadratic or threshold-like patterns that reflect plant adaptation strategies and trade-offs along environmental gradients^[10,11]^.

In addition to latitude, climate, soil, and forest structural factors are also important drivers of spatial variation in leaf traits. Climate acts as a primary constraint on plant function at macroecological scales by regulating temperature regimes, water availability, and solar radiation. These climatic controls shape plant physiological processes, metabolic rates, and photosynthetic capacity, thereby influencing leaf trait expression^[12,13]^. Temperature and precipitation jointly define the thermal and hydric framework limiting plant growth: in warm and humid regions, higher photosynthetic rates and nutrient cycling favor the development of thin, nitrogen-rich leaves^[14]^, whereas in cold and arid regions, plants tend to produce thick leaves with high LDMC to enhance tissue toughness and drought resistance^[15]^. Soil factors influence plant traits and stress adaptation through nutrient availability and chemical properties. In many terrestrial ecosystems, nitrogen and phosphorus are the primary limiting nutrients, and their availability strongly influences leaf N and leaf P concentrations and their stoichiometric balance^[16,17]^. Soil pH indirectly affects nutrient uptake and utilization efficiency by regulating nutrient forms and microbial activity^[18]^. In nutrient-poor soils of arid or high-latitude regions, plants often adapt by enhancing nutrient use efficiency or producing high-LDMC leaves, consistent with the 'nutrient limitation hypothesis', which has been validated across multiple ecosystems^[19,20]^. Forest structural factors reflect the combined influence of community structure and biotic interactions. Forest age indicates successional stage and historical resource accumulation, with trait differences among ages reflecting dynamic trade-offs between growth and conservation strategies^[21]^. Forest density affects light distribution, competition intensity, and water use, thereby shaping leaf traits; for example, individuals in high-density stands often increase SLA or reduce LDMC to enhance light capture efficiency^[22,23]^. Species richness represents community diversity and determines the range and community-weighted mean (CWM) of functional traits, ultimately influencing ecosystem functional stability and nutrient use efficiency^[24]^. Such diversity differences between forest types are especially relevant because they influence community functional composition and modulate how leaf traits respond to climate, soil conditions, and stand structure^[25]^. Although previous studies have highlighted these drivers, most have focused on local or regional scales and considered the effects of single environmental factors on traits. Few studies have integrated climate, soil, and forest structure at a national scale. In complex environments such as arid, cold, or transitional ecological zones, climatic, edaphic, and stand structural factors often interact rather than acting independently, jointly shaping plant resource-use strategies and leaf traits^[26]^. Therefore, there is an urgent need to disentangle the relative contributions and pathways through which multiple environmental drivers shape spatial variation in leaf traits at macroecological scales.

Meanwhile, the ecological response differences between natural and planted forests have received increasing attention. Natural forests typically undergo long-term succession, resulting in higher species diversity, greater structural complexity, and more stable community functional states shaped by prolonged environmental filtering^[27]^. Their leaf traits often represent conservative ecological strategies—such as higher dry matter investment and lower specific leaf area—reflecting long-term evolutionary acclimation and functional optimization to relatively stable environmental conditions^[28]^. In contrast, planted forests are shaped by anthropogenic interventions, including afforestation, thinning, fertilization, and species selection, which lead to simplified stand structures, younger stand ages, and reduced community complexity. Consequently, leaf traits in planted forests tend to show higher environmental plasticity and weaker long-term trait coordination relative to natural forests^[29,30]^. Previous studies in Chinese forest ecosystems have reported substantial spatial variation in key leaf traits along climatic and edaphic gradients: SLA generally decreases toward colder or less favorable environments^[31]^, while LDMC and structural investment increase with environmental stress, reflecting shifts from acquisitive to conservative strategies^[32]^. In planted forests, high trait plasticity has been widely documented, driven by stand age, density, and management intensity, with fast-growing species typically showing higher SLA and nutrient concentrations than those in natural forests^[33]^. However, most existing studies are restricted to specific regions, single forest types, or narrow environmental gradients, and few have simultaneously compared natural and planted forests across broad latitudinal zones using integrative climate–soil–stand frameworks. This knowledge gap limits our understanding of large-scale differences in trait–environment coupling, and constrains predictions of forest functional dynamics and the ecological consequences of management strategies under environmental change.

Building on the above knowledge gaps, this study aims to quantify broad-scale latitudinal patterns of four key leaf functional traits (SLA, LDMC, LN, LP) in natural and planted forests across China and to identify the dominant environmental drivers shaping these patterns. Specifically, we seek to: (i) determine whether natural and planted forests exhibit distinct latitudinal trends in leaf morphological and nutrient traits; (ii) disentangle the relative contributions of climate, soil, and stand structure to trait variation across forest types; and (iii) clarify the direct and indirect pathways through which latitude regulates leaf traits. These objectives establish an integrated framework for assessing differences in trait–environment coupling and evaluating the functional stability of natural versus planted forests across broad environmental gradients. To address these objectives, latitudinal variation was analyzed in SLA, LDMC, LN, and LP using 482 forest plots across China (105 natural and 377 planted forests), surveyed between 2008 and 2020. Non-monotonic trait–latitude relationships were quantified using quadratic polynomial models, and the relative importance of climatic, soil, and stand structural factors evaluated through correlation analysis, variance partitioning, machine-learning–based feature importance assessment, and structural equation modeling (SEM). Because climate and soil constraints do not change linearly with latitude—showing mid-latitude climatic optima, nutrient thresholds, and shifts between acquisitive and conservative strategies—leaf functional traits frequently respond in nonlinear or unimodal patterns along large-scale geographic gradients. Based on previous large-scale trait–climate analyses and evidence for management-induced trait plasticity, three explicit, testable hypotheses are proposed: (i) Leaf functional traits show non-monotonic latitudinal patterns (U- or inverted U-shaped), especially for traits associated with climatic optima (e.g., SLA, LP); (ii) because management regulates resource availability and stand structure, planted forests are expected to show stronger trait sensitivity to stand variables (age, density, species richness) and to latitude compared with natural forests; (iii) the effects of latitude on leaf traits occur primarily through indirect climatic and edaphic pathways (latitude → climate → soil → stand structure → traits), with the strength of these pathways differing between natural and planted forests. Understanding these mechanisms has important implications for forest management. For natural forests, identifying trait–environment relationships can inform conservation strategies that maintain structural complexity and long-term functional stability under climate change. For planted forests, clarifying how stand structure and management practices regulate leaf traits can guide the optimization of planting density, species composition, and resource inputs to enhance productivity, resilience, and carbon sequestration.

## Materials and methods

### Sample data

A total of 482 forest plots were included in the present study, comprising 246 plots from original field surveys, and 236 plots compiled from published literature. This study integrated field survey data and literature sources collected between 2008 and 2020, covering forest ecosystems across different climatic zones, and vegetation types in China. A total of 482 forest plots were obtained, including 377 planted forests, and 105 natural forests. Natural forests were defined as forest communities that have regenerated naturally without artificial planting, typically characterized by complex canopy structures, high species diversity, and minimal anthropogenic disturbance. Planted forests were defined as stands established through afforestation or reforestation, generally composed of one or a few tree species and subject to management interventions such as thinning, fertilization, or pruning. The data for all planted and natural forest plots are provided in Supplementary Data 1.

Plot selection followed three criteria: (i) forest type was clearly defined (natural or planted); (ii) complete information on forest structure and environmental conditions was available; and (iii) plots were representative of regional vegetation and climate characteristics. To ensure consistency and comparability between the field and literature data, the following procedures were applied: strict inclusion criteria for literature data were adopted, including only studies reporting the same forest types, geographic locations, and leaf traits (SLA, LDMC, LN, LP) measured following standardized protocols. All literature data were carefully checked and converted to match the units, measurement methods, and trait definitions used in the field surveys. Environmental and stand variables (e.g., climate, soil, forest structure) from literature sources were also verified for compatibility with field measurements, and any outliers or inconsistent entries were excluded. Through these steps, the combined dataset of 482 plots is comparable and reliable for analyzing latitudinal patterns of leaf traits.

At each site, four adjacent 30 × 30 m subplots were established, avoiding areas with obvious human disturbance. GPS devices were used to record latitude, longitude, and elevation. Forest structural factors included forest age, forest density, and species richness. Fertilization is not routinely applied in the studied forest types, and no plots had documented fertilization records in the inventory datasets used in this study. Therefore, fertilization was not included as a stand-level variable. Forest age and management history were obtained through field surveys, interviews, and literature sources. For planted forests, stand age was obtained from local forestry bureaus and management records, where planted forests establishment years are explicitly documented. For natural forests, forest age was taken from official stand-age descriptions provided by local forestry and resource management agencies during field surveys. These records reflect regionally recognized stand developmental stages, rather than precise tree ages, and are the most reliable stand-level age information available for large-scale analyses. This standardized classification is widely used in regional forest ecological studies. Species richness was recorded at the plot level, and used as a structural variable; species identities were not required for community-level trait analyses. Forest density was quantified as the number of trees per unit area, which, together with stand age and species richness, provides an effective representation of key components of stand structure in large-scale forest assessments. In this study, stand age, stand density, and species richness were focused on as forest structural variables, as these metrics are consistently defined across large-scale forest inventories and literature sources, and they effectively capture the dimensions of stand structure that are relevant to leaf trait variation at the national scale. Other metrics (e.g., canopy height, basal area) may enrich local-scale analyses but are not essential for the present cross-regional comparisons.

### Functional trait data

Four representative leaf functional traits—specific leaf area (SLA), leaf dry matter content (LDMC), leaf nitrogen content (LN), and leaf phosphorus content (LP)—were selected to reflect plant strategies for resource acquisition, utilization, and conservation. Within each plot, 3–5 dominant tree species were sampled. While species were selected based on dominance in the canopy, their identities were not documented, consistent with the focus on plot-averaged functional traits. For each species, 3–5 healthy, mature individuals were selected, and 5–10 fully expanded sun-exposed leaves were collected per individual during July–August to ensure phenological consistency. Leaf area was measured using a scanner (Canon CanoScan LIDE 110). Leaf fresh mass was measured immediately after harvesting using a high-precision electronic balance (0.0001 g), with particular care taken to minimize the time between leaf excision and weighing to limit water loss. Given the field-based sampling conditions, leaf fresh mass was determined directly after harvest without a rehydration step^[34]^. After weighing, leaves were heated at 105 °C for 15 min to deactivate enzymes, and then oven-dried at 60 °C for 48–72 h until reaching constant mass. SLA (m^2^/kg) was calculated as the ratio of leaf area to dry mass, and LDMC (g/g) as the ratio of dry mass to fresh mass. Leaf nitrogen content was measured using an elemental analyzer, while leaf phosphorus content was determined by the molybdenum–antimony anti-colorimetric method. All measurements followed the standardized protocols proposed by Cornelissen et al.^[35]^, to ensure comparability across forest types^[32]^. At the plot scale, community-weighted mean (CWM) leaf trait values were calculated by weighting species-level mean traits by their relative abundance within each plot, thereby providing an integrated representation of community-level functional composition relevant to this macroecological analysis.

### Environmental data

Climatic variables included mean annual temperature (MAT), mean annual precipitation (MAP), mean annual evapotranspiration (MAE), and annual sunshine duration (ASD), obtained from the WorldClim database (www.worldclim.org, 1 km resolution), and the China Meteorological Data Service Center (http://data.cma.cn). Soil variables were collected from the national soil databases (http://soil.geodata.cn; www.osgeo.cn/data/wc137, 250 m resolution) and included pH, total nitrogen (Soil N), and total phosphorus (Soil P) for the 0–30 cm soil layer. Spatial distribution maps of the plots were generated using ArcMap (v10.8), and climate and soil data were matched to each plot's GPS coordinates using bilinear interpolation implemented via the raster and terra packages in R, ensuring high spatial accuracy of environmental variables. Forest structural variables included forest age, forest density (trees ha^−1^), and species richness (number of tree species per plot).

### Data analysis

All statistical analyses were conducted in the R environment (v4.3.1, R Core Team, 2023). First, the spatial distribution of the 482 forest plots across China was visualized using ArcMap 10.8. Plot coordinates recorded via GPS were overlaid with China's administrative boundary vector data (1:1,000,000) to generate distribution maps for natural and planted forests. These maps provided a visual reference for latitudinal and climatic distribution patterns and guided subsequent trait–environment analyses.

To examine latitudinal patterns of leaf functional traits, quadratic polynomial regression models were fitted separately for SLA, LDMC, LN, and LP in natural and planted forests:

$ \mathrm{Trait}={\beta }_{0}+{\beta }_{1}(\mathrm{Latitude})+{\beta }_{2}({\mathrm{Latitude}}^{2})+\varepsilon $

where, Trait represents the leaf functional trait; *β*_0_ is the intercept, reflecting the estimated trait value at latitude zero; *β*_1_ is the linear term coefficient, indicating the linear trend of trait variation with latitude (*β*_1_ > 0 indicates increasing trait values with latitude, and vice versa); *β*_2_ is the quadratic term coefficient, representing the nonlinear response to latitude (*β*_2_ > 0 indicates a U-shaped pattern with lowest values at mid-latitudes, while *β*_2_ < 0 indicates an inverted-U pattern with highest values at mid-latitudes, reflecting an optimal functional zone or inflection point); and *ε* is the residual error. Model fit was assessed using R^2^, and significance was determined at *p* ≤ 0.05. This approach allowed evaluation of both linear and nonlinear trends in leaf traits along latitudinal gradients and comparison of responses between forest types.

Pearson correlation coefficients between leaf traits and environmental variables were calculated using the linkET package in R, and correlation heatmaps were plotted. Climate variables included MAT, MAP, MAE, and ASD; soil variables included Soil N, Soil P, and Soil pH; forest structural variables included forest age, forest density, and species richness. Significance was tested using two-tailed tests (* *p* < 0.05; ** *p* < 0.01; *** *p* < 0.001).

To quantify the relative contributions of climate, soil, and forest factors to spatial variation in leaf traits, hierarchical partitioning analysis was performed using the rdacca.hp package. This method separates independent and joint contributions of environmental factors, identifying the primary drivers in natural versus planted forests. Linear response strength was visualized via barplots of standardized regression coefficients.

Further, the CatBoost machine learning algorithm (Category Boosting) was applied to rank the importance of environmental factors. SLA, LDMC, LN, and LP served as response variables, while climate, soil, and forest variables were predictors. Five-fold cross-validation was used to reduce overfitting, and feature importance scores quantified the independent contribution of each variable. Permutation tests (*n* = 1,000) assessed the significance of differences in feature importance (*p* < 0.05).

Finally, piecewise structural equation modeling (piecewiseSEM) was used to examine direct and indirect effects of latitude, climate, soil, and forest factors on leaf traits. All component equations in the piecewise SEM were fitted using linear models (lm), resulting in a linear structural equation model. In this study, it was considered that the response of leaf traits in planted forests and natural forests to latitude exhibits a quadratic polynomial relationship. To satisfy the assumption of linear relationships in structural equation modeling (SEM), a square-root transformation was performed on the dependent variable of leaf traits to approximate its linearity with the independent variables (i.e., presenting a linear relationship in one variable). Subsequently, a linear SEM was constructed based on this to conduct path analysis, ensuring that the model assumptions were met. This allowed the reliable assessment of the direct and indirect effects of climate, soil, and stand factors on leaf traits and to quantify the multi-factor interactions at a large spatial scale. Linear models (lm) were used for each path, and standardized path coefficients quantified effect strength and direction. Model fit was evaluated using Fisher's C statistic, and paths were considered significant at *p* < 0.05. Separate SEMs were constructed for natural and planted forests to compare structural differences in trait formation mechanisms. Path diagrams illustrated the direction and magnitude of effects (arrow direction indicates positive or negative effect, line thickness reflects effect strength). In addition to estimating path coefficients, total effects were decomposed into direct and indirect components to quantify the mediated influence of latitude, climate, soil properties, and forest structure on leaf traits. This linear formulation follows the standard implementation of the piecewiseSEM framework, and is widely used in ecological applications. Because the goal of the SEM is to quantify direct and indirect pathways rather than model detailed non-monotonic functional forms, linear paths provide an appropriate and reliable approximation for evaluating multi-factor interactions at broad spatial scales. Multicollinearity was assessed via variance inflation factors (VIF), retaining variables with VIF < 10. All significance tests were two-tailed (*α* = 0.05), and multiple comparisons were corrected using the Benjamini–Hochberg method.

## Results

**Figure 1:**
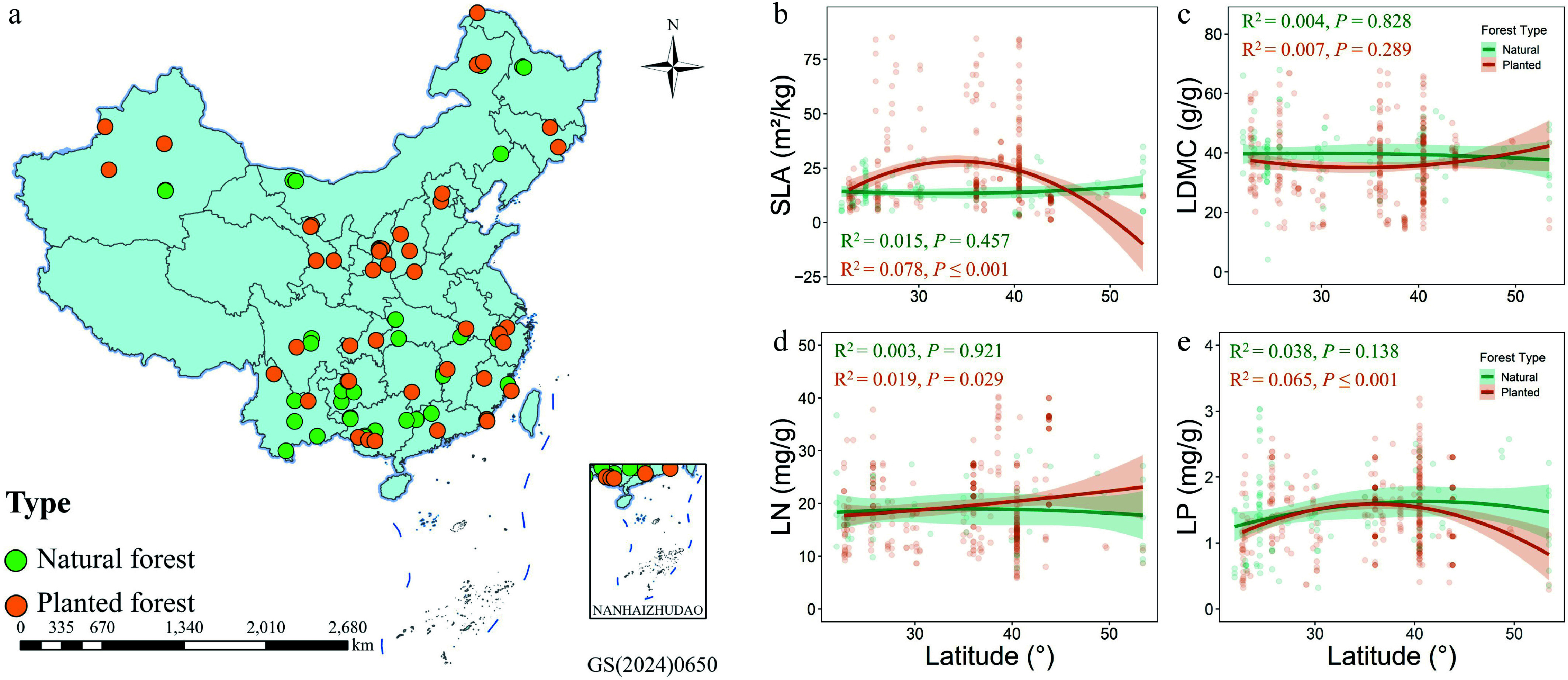
(a) Spatial distribution of natural forests (green), and planted forests (orange) sampling sites; (b)–(e) Latitudinal variation patterns of specific leaf area (SLA), leaf dry matter content (LDMC), leaf nitrogen (LN), and leaf phosphorus (LP) in the two forest types.

Specific leaf area (SLA) in planted forests exhibited a significant quadratic relationship with latitude (*p* ≤ 0.001), with higher values at mid-latitudes, and lower values at low and high latitudes, indicating strong plasticity of leaf structure along the latitudinal gradient. Leaf dry matter content (LDMC) showed no significant latitudinal response in either forest type (*p* > 0.05), suggesting low sensitivity of LDMC to latitude (Fig. 1c). Leaf nitrogen content (LN) slightly increased with latitude in planted forests (*p* = 0.029) but showed no significant trend in natural forests, implying that planted forests at higher latitudes may enhance nitrogen accumulation or optimize nutrient-use strategies to cope with lower temperatures (Fig. 1d). Leaf phosphorus content (LP) also displayed a significant quadratic relationship with latitude in planted forests (*p* ≤ 0.001), whereas no significant response was observed in natural forests (Fig. 1e). Overall, leaf traits in planted forests responded more strongly to latitudinal gradients than in natural forests, indicating higher environmental plasticity, while natural forests exhibited greater homeostatic stability.

**Figure 2:**
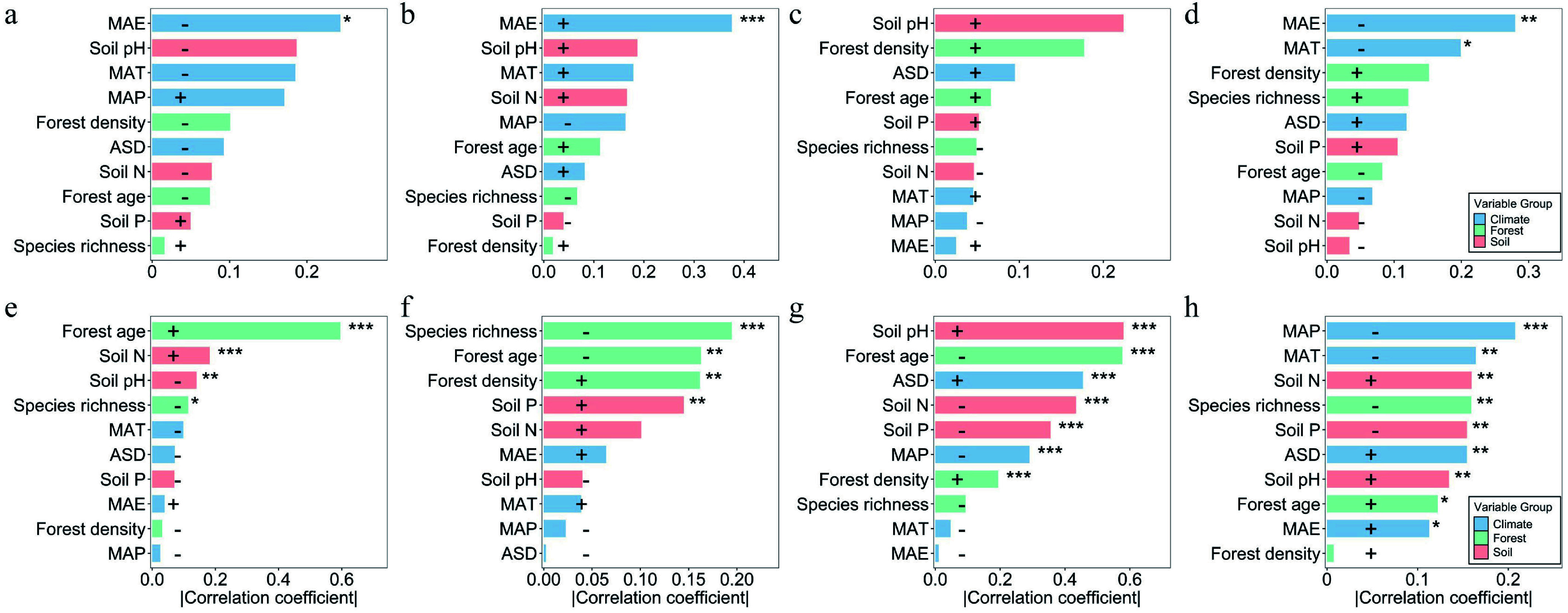
Correlation analysis between leaf traits and environmental factors. Pearson correlation coefficients between leaf traits and climate, soil, stand structure, and latitude are shown for natural and planted forests. (a)–(d) represent natural forests, and (e)–(h) represent planted forests. Blue, pink, green, and orange bars correspond to climate, soil, forest structure, and latitude factors, respectively; bar length indicates the magnitude of the correlation coefficient, and the direction (positive or negative) indicates the correlation sign. Significance levels: *** *p* < 0.001; ** *p* < 0.01; * *p* < 0.05. SLA: specific leaf area; LDMC: leaf dry matter content; LN: leaf nitrogen concentration; LP: leaf phosphorus concentration.

Environmental driver analysis revealed distinct trait–environment response patterns between forest types. In natural forests, SLA, LDMC, and LP were significantly correlated only with mean annual evapotranspiration (MAE): SLA and LP were negatively correlated, whereas LDMC was positively correlated; LN showed no significant response to environmental factors (Fig. 2a–d). In contrast, leaf traits in planted forests were significantly influenced by multiple environmental factors. Forest age had a prominent effect, positively correlated with SLA and LP, but negatively with LDMC and LN. SLA, LN, and LP were negatively correlated with species richness, suggesting that higher community complexity may constrain leaf trait development in planted forests. Soil nutrients also played a key role: Soil N was negatively associated with SLA, LN, and LP; Soil P was negatively associated with LN and LP, but positively with LDMC. LN and LP were additionally modulated by climate variables (Fig. 2e–h).

**Figure 3:**
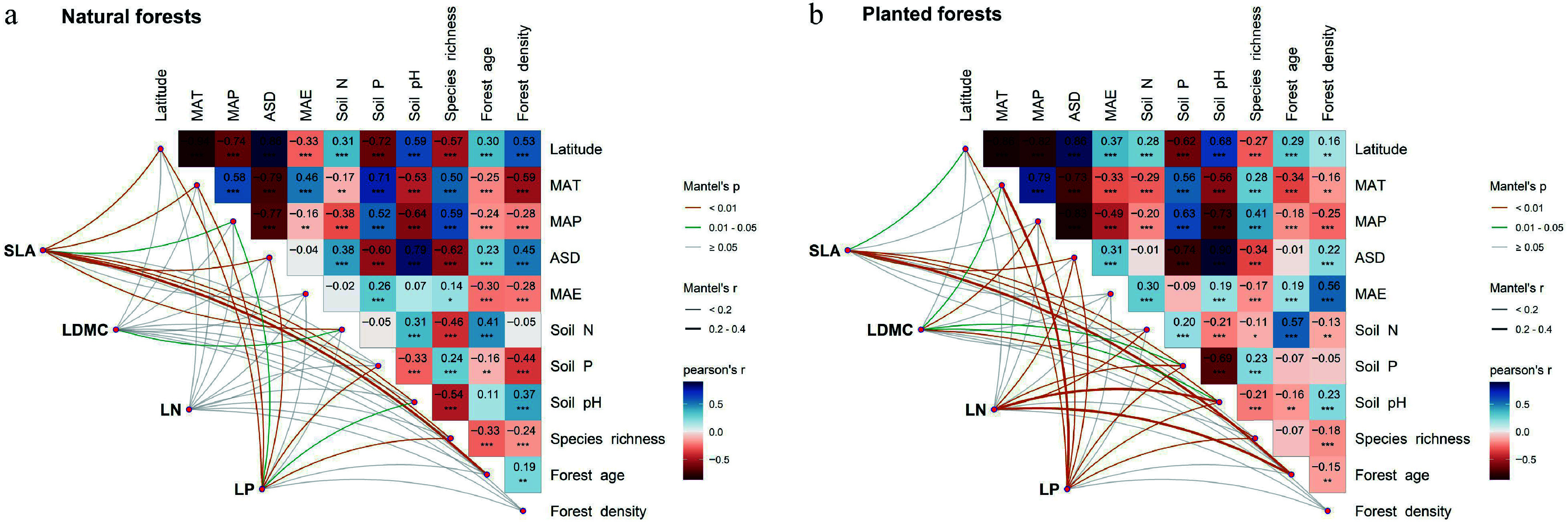
Multiple correlation analysis of potential drivers influencing leaf traits in (a) natural, and (b) planted forests. Climate factors include mean annual temperature (MAT), mean annual precipitation (MAP), mean annual evapotranspiration (MAE), and annual sunshine duration (ASD); soil factors include soil total nitrogen (N), available phosphorus (P), and soil pH; forest factors include forest age, forest density, and species richness. Significance levels: *** *p* < 0.001; ** *p* < 0.01; * *p* < 0.05.

**Figure 4:**
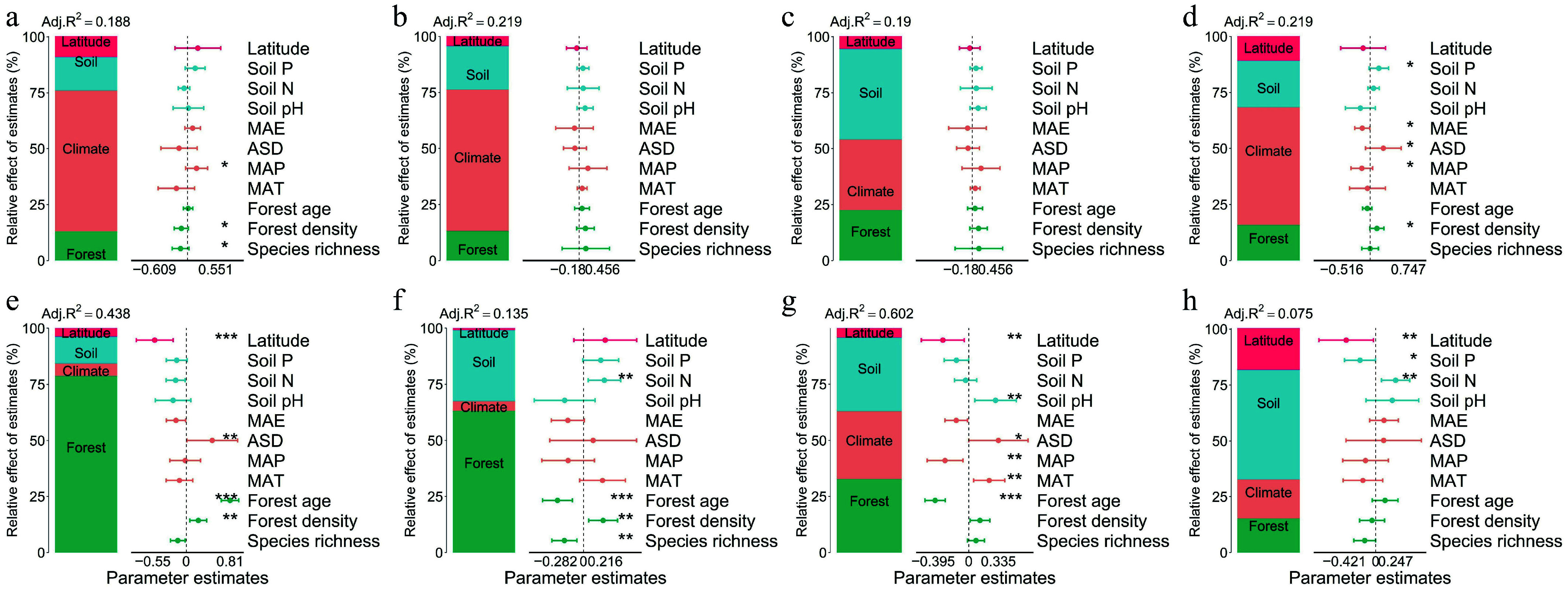
Relative contributions of environmental and stand structural variables to geographic variation in leaf functional traits (SLA, LDMC, LN, and LP) in natural and planted forests. Each panel consists of two sub-panels. The left sub-panel shows the partitioning of explained variance among four predictor groups—Latitude, Soil (Soil N, Soil P, Soil pH), Climate (MAT, MAP, MAE, ASD), and Forest structure (species richness, forest age, forest density)—based on hierarchical partitioning using adjusted R^2^ from redundancy analysis (RDA). The right sub-panel presents standardized regression coefficients (± SE), quantifying the direction and magnitude of individual predictors. Positive values indicate positive effects, and negative values indicate negative effects. A broken-axis transformation was applied where necessary to visualize both small and large coefficient estimates simultaneously. Filled points are colored by predictor category (Forest, Climate, Soil, Latitude), significance levels: *** *p* < 0.001; ** *p* < 0.01; * *p* < 0.05. Panel descriptions: Natural forests: (a) SLA, (b) LDMC, (c) LN, (d) LP; Planted forests: (e) SLA, (f) LDMC, (g) LN, (h) LP.

Integrative analyses of climate, soil, and forest factors demonstrated significant differences in environmental response mechanisms between natural and planted forests (Fig. 3). Variance partitioning and multiple linear regression showed that leaf traits in natural forests were primarily jointly influenced by climate and soil, with latitude exerting relatively weak direct effects (Fig. 4a–d). Conversely, leaf traits in planted forests responded strongly to latitude, especially SLA, LN, and LP. Latitude contributed most to LP variation in planted forests, exceeding the influence of climate and forest factors. Specifically, SLA (78.7%) and LDMC (63.11%) were primarily driven by forest factors; LN was influenced by forest (32.71%), climate (30.45%), and soil (32.79%) jointly; LP was mainly driven by soil (49.07%), followed by latitude (18.67%), climate (17.47%), and forest (15.20%) (Fig. 4e–h).

**Figure 5:**
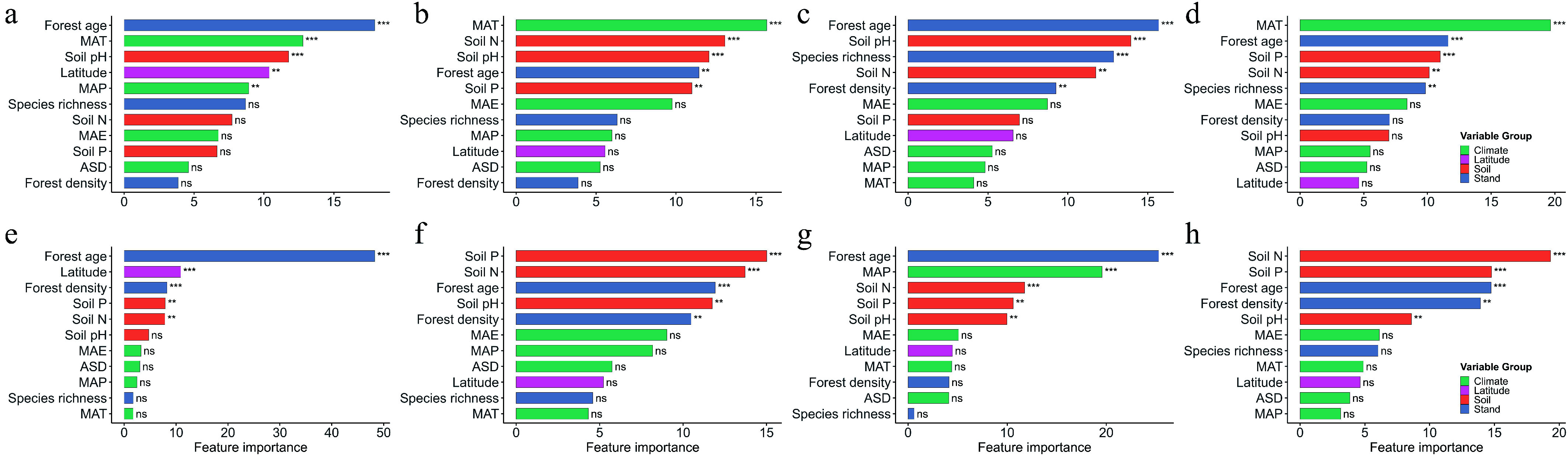
Relative importance of environmental and stand structural predictors for leaf functional traits in natural and planted forests based on CatBoost models. Results for natural forests—(a) SLA, (b) LDMC, (c) LN, and (d) LP—and planted forests—(e) SLA, (f) LDMC, (g) LN, and (h) LP. Predictor variables were grouped into four categories: Climate (MAT, MAP, ASD, MAE), Soil (Soil pH, Soil N, Soil P), Stand structure (species richness, forest age, forest density), and Latitude. Feature importance was derived from CatBoost using 300 iterations and RMSE loss. Bars represent the relative contribution of each predictor, with colors indicating predictor groups. Asterisks mark relative importance ranks (*** top 3, ** top 4–5, ns = not ranked), highlighting the most influential predictors for each trait. Higher bar height indicates stronger predictive contribution to trait variation.

Feature importance analysis further highlighted forest age as a key determinant of all four leaf traits in both forest types. Latitude also played a prominent role in explaining SLA variation across both forests. In natural forests, climate factors were among the top predictors for SLA, LDMC, and LP, except for LN. In planted forests, soil nitrogen and phosphorus were consistently important across all traits, and forest density significantly influenced all traits except LN (Fig. 5).

**Figure 6:**
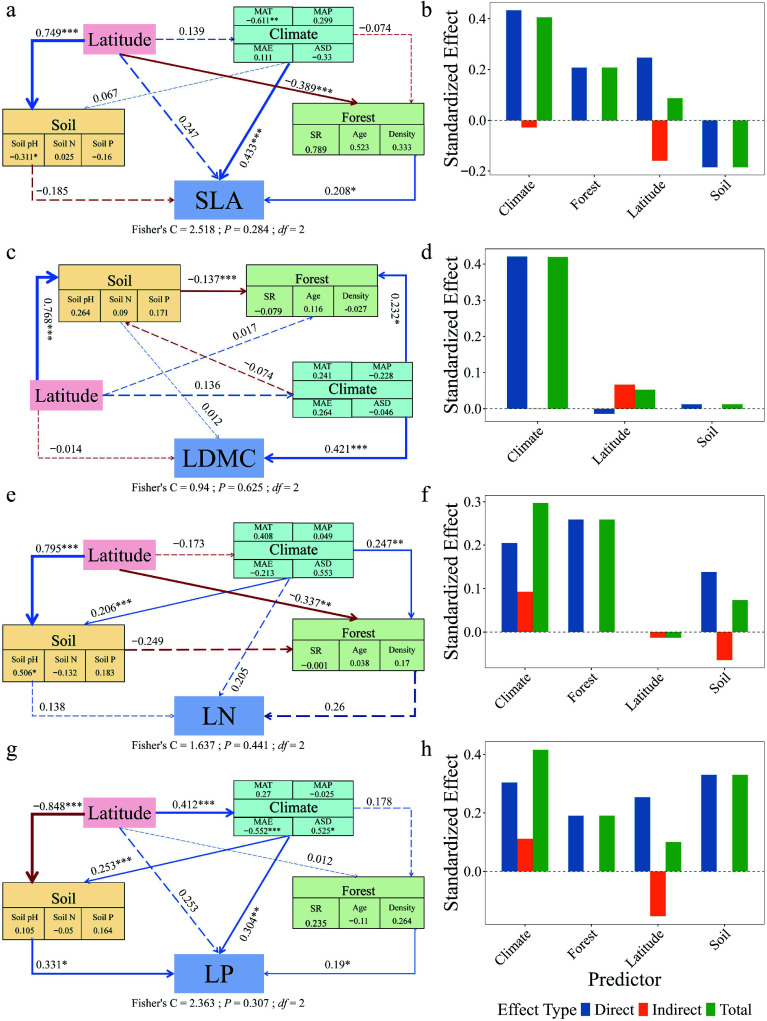
Structural equation models (SEMs) and effect decomposition of leaf trait responses to latitude in natural forests. (a), (c), (e), (g) Represent SEMs for specific leaf area (SLA), leaf dry matter content (LDMC), leaf nitrogen concentration (Leaf N), and leaf phosphorus concentration (Leaf P), respectively. (b), (d), (f), (h) Show the decomposition of standardized direct effects (blue), indirect effects (orange), and total effects (green) of environmental factors on leaf traits. Solid blue arrows indicate significant positive paths, solid red arrows indicate significant negative paths, and arrow thickness represents the magnitude of standardized path coefficients. Dashed arrows denote non-significant paths. Numbers adjacent to arrows indicate standardized path coefficients for significant paths only (* *p* < 0.05; ** *p* < 0.01; *** *p* < 0.001). Fisher's C statistic is shown as a measure of model fit.

**Figure 7:**
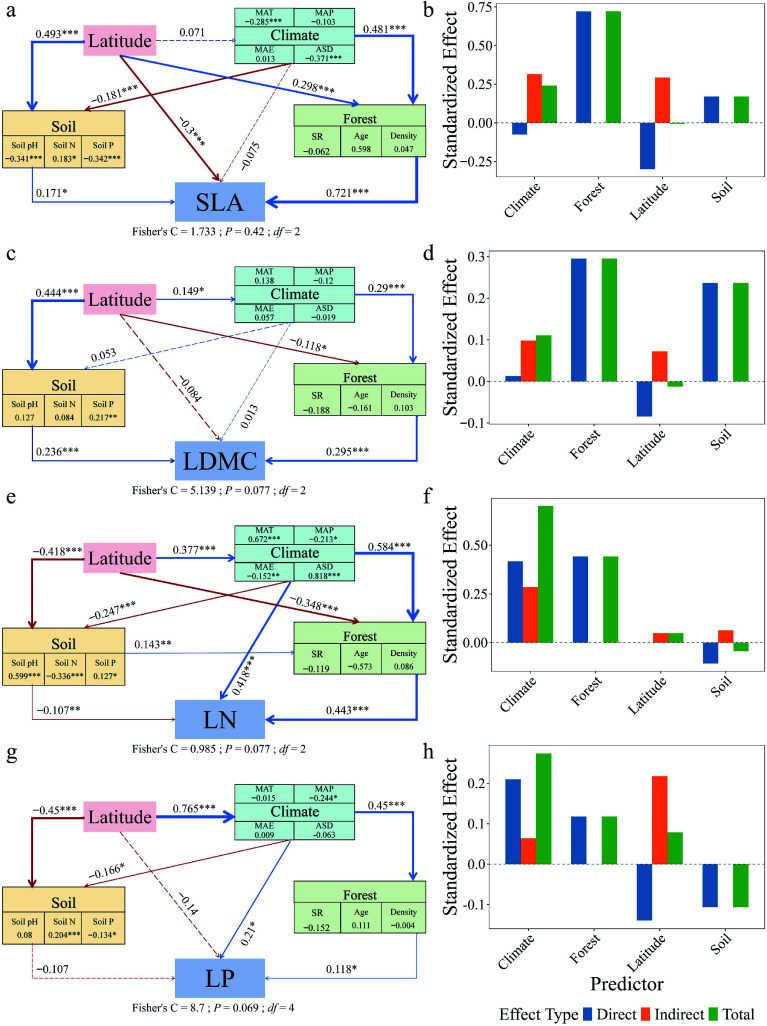
Structural equation models (SEMs) and effect decomposition of leaf trait responses to latitude in planted forests. (a), (c), (e), (g) Represent SEMs for specific leaf area (SLA), leaf dry matter content (LDMC), leaf nitrogen concentration (Leaf N), and leaf phosphorus concentration (Leaf P), respectively. (b), (d), (f), (h) Show the decomposition of standardized direct effects (blue), indirect effects (orange), and total effects (green) of environmental factors on leaf traits. Solid blue arrows indicate significant positive paths, solid red arrows indicate significant negative paths, and arrow thickness reflects the magnitude of standardized path coefficients. Dashed arrows denote non-significant paths. Numbers adjacent to arrows indicate standardized path coefficients for significant paths only (* *p* < 0.05; ** *p* < 0.01; *** *p* < 0.001). Fisher's C statistic is presented as a measure of model fit.

Piecewise structural equation modeling revealed contrasting paths of multi-factorial influence on leaf traits between forest types (Figs 6, 7). In natural forests, SLA (path coefficient = 0.247), LDMC (–0.014), and LP (0.253) were directly influenced by latitude. In planted forests, SLA (–0.300), LDMC (–0.084), and LP (–0.140) were also directly influenced by latitude, but with opposite directions. LN in both forest types was primarily driven by forest factors, with indirect modulation via climate. Decomposition of the SEM pathways showed that latitude influenced leaf traits both directly and indirectly through its effects on climate, soil nutrients, and forest structure, with these mediated pathways constituting a substantial portion of the total effects (Figs 6, 7). Planted forests exhibited higher sensitivity and response magnitude under these multi-factor regulatory mechanisms, reflecting stronger plasticity, and adaptive capacity of leaf functional traits to environmental gradients.

## Discussion

At the national scale, this study systematically compared spatial variation patterns of leaf functional traits along latitudinal gradients, and their multidimensional environmental drivers in natural and planted forests. The results demonstrate pronounced divergence in the responses of leaf morphological and nutrient traits to latitude, climate, soil, and forest factors between the two forest types. Natural forests exhibited strong functional homeostasis and consistent environmental adaptation, whereas planted forests showed higher plasticity and greater sensitivity to environmental variation. These differences reflect the distinct pathways through which long-term natural selection and anthropogenic management shape plant functional strategies, highlighting the potential trade-offs between ecosystem stability and the intensity of human intervention.

### Differentiation of leaf functional traits along latitudinal gradients and their ecological adaptation mechanisms

The present study revealed that specific leaf area (SLA), and leaf phosphorus content (LP) in planted forests exhibited significant quadratic relationships with latitude, whereas changes in natural forests were comparatively muted (Fig. 1b–e). This indicates that leaf structure and function in planted forests are more responsive to geographic environments, reflecting higher phenotypic plasticity. These quadratic patterns may partly reflect shifts in community composition or dominant-species contributions along the latitudinal gradient. However, species-level trait and phylogenetic data were not consistently available across the literature-based and field-survey datasets used in this study, and community-level structural variables (stand age, density, and richness) were therefore used as integrative proxies. Species-specific mechanisms remain an important direction for future research. SLA, a key indicator of photosynthetic potential and resource acquisition capacity, represents the resource-acquisitive end of the leaf economics spectrum^[36,37]^. The pattern of elevated SLA at mid-latitudes, and reduced values at both low and high latitudes in planted forests suggests that in climatically moderate and sufficiently wet mid-latitude regions, planted species invest in a larger photosynthetic area to achieve rapid growth^[38]^. In contrast, resource limitations in cold high-latitude, and hot low-latitude regions drive a shift toward conservative strategies, resulting in lower SLA. This reflects adaptations to thermal stress, nutrient constraints, or competition in warmer low-latitude environments, which are often humid but seasonally variable in moisture^[39]^. This non-monotonic response supports the 'optimal climatic niche hypothesis', in which leaf traits exhibit functional optima along environmental gradients^[40]^. In natural forests, the lack of a significant latitudinal response in SLA indicates that long-term species replacement and community assembly have produced a stable functional structure. This stability reduces the sensitivity of SLA to external climatic gradients, reflecting a strong ecological buffering capacity^[41,42]^.

Leaf dry matter content (LDMC) did not show significant latitudinal variation in either forest type, yet its patterns were strongly linked to stand structural attributes and broader environmental conditions. This indicates that variation in leaf mass investment—a key component of the leaf economics spectrum associated with leaf durability—is jointly governed by stand structure, competitive dynamics^[43]^. Higher LDMC is generally linked to enhanced drought resistance, prolonged tissue longevity, and conservative resource-use strategies^[44,45]^. In dense or older stands, individuals increase tissue density to withstand light competition and drought stress, a trend reflected in variance partitioning results, with forest factors explaining up to 63.1% of LDMC variation in planted forests. In natural forests, LDMC was positively correlated with mean annual evapotranspiration (MAE), suggesting that in areas with higher evaporative demand, plants tend to develop structurally dense, long-lived leaves to maintain carbon balance and water stability. This pattern aligns with the 'drought-adaptive conservative strategy', indicating that long-term environmental filtering can shape trait stability at the community level^[46,47]^. The present analysis relies on plot-level CWM trait values aggregated from all sampled species, without considering intraspecific variation. This community-aggregated approach is appropriate for capturing broad-scale functional patterns across large environmental gradients, but it does not allow the attribution of trait variation to specific taxa, or species-level strategies. Consequently, species-specific adaptations and phylogenetic influences may not be fully resolved. Future studies that integrate species identity and abundance information would enable a more detailed assessment of taxonomic and phylogenetic contributions to trait–environment relationships.

### Synergistic regulation of leaf functional traits by climate and soil

Climate and soil constitute the two primary environmental axes shaping the spatial patterns of leaf functional traits. The present results indicate that leaf traits in natural forests are predominantly driven by the combined effects of climate and soil, whereas in planted forests, forest structure, and latitude play more prominent roles. Climatic factors directly influence photosynthetic rate, transpiration balance, and energy-use efficiency by modulating temperature and precipitation regimes^[48]^, while soil factors determine the availability of nutrients and the rate of rhizosphere nutrient transformation^[49]^. In natural forests, SLA, LDMC, and LP were significantly correlated with mean annual evapotranspiration (MAE), highlighting the dominant role of climate in shaping trait orientation under long-term natural selection (Fig. 2a–d). Climatic regulation typically operates by affecting leaf metabolic activity, enzymatic reaction rates, and the stability of the photosynthetic apparatus^[50]^. Within the framework of the leaf economics spectrum (LES), plants exhibit coordinated trait syndromes reflecting trade-offs between rapid resource acquisition and conservation. In warm and humid environments, plants tend to produce 'resource-acquisitive' leaves characterized by high specific leaf area (SLA), and elevated leaf nitrogen content (LN), which facilitate rapid photosynthetic rates, high growth potential, and a fast plant life-history strategy^[51]^. Conversely, in cold or drought-prone environments, plants invest in 'resource-conservative' leaves with high leaf dry matter content (LDMC), and low SLA, optimizing structural stability, nutrient retention, and longevity, consistent with a slow life-history strategy^[52]^. These patterns illustrate that latitudinal gradients in climate select for distinct leaf trait syndromes that mediate ecological performance and community assembly across forest ecosystems.

Soil nutrient availability exhibited distinct effects between the two forest types (Fig. 2). In planted forests, soil nitrogen (Soil N) was negatively correlated with LN, LP, and SLA, indicating that higher total soil nitrogen does not necessarily translate into increased leaf nutrient content. This pattern likely reflects differences in planted forests establishment history and species selection, which can influence soil nutrient dynamics, and leaf nitrogen uptake strategies^[53]^. Although stand-level management records were not available across all data sources, the large-scale and multi-source nature of our dataset provides a robust basis for identifying community-level soil–leaf nitrogen relationships. The specific roles of management legacy and species composition warrant further investigation in studies with detailed stand-level documentation. Additionally, soil phosphorus (Soil P) was positively associated with LDMC, but negatively with LN and LP, suggesting that in phosphorus-limited sites, plants enhance tissue density and longevity to maintain long-term nutrient-use efficiency^[54,55]^. In natural forests, these relationships were milder, indicating that leaf traits and soil nutrients have reached a dynamic equilibrium through long-term successional processes. Overall, climatic factors exert more pronounced direct effects on leaf traits, while soil influences traits indirectly by modulating nutrient uptake and energy allocation. The interaction of climate and soil ultimately governs the spatial differentiation of leaf functional traits^[32,56]^.

### Modulatory role of forest structure

Forest-level factors serve as critical intermediaries linking environmental conditions with community functional structure. The present results indicate that in planted forests, forest age and density contribute significantly more to leaf trait variation than in natural forests. Feature importance ranking from CatBoost analysis revealed that forest age consistently ranked among the top five predictors across all leaf traits, highlighting the pervasive regulatory role of community age structure in shaping functional traits (Fig. 5).

In young stands, plants tend to adopt fast-growth strategies characterized by high SLA and low LDMC, facilitating rapid resource acquisition and canopy occupation. Conversely, older stands exhibit conservative strategies with high LDMC and low SLA, maintaining ecological balance and structural stability^[57]^. Forest age in natural forests was obtained from official inventory-based stand-age classes, reflecting developmental stages rather than the exact ages of individual trees. This standardized classification is widely used in large-scale assessments, ensuring comparability at the national level. High-density stands intensify competition, promoting thinner leaves to maximize light capture, whereas in sparse stands, thicker leaves enhance water retention and defensive capacity^[58,59]^. In planted forests, low-diversity planting schemes and density-dependent competition shape both horizontal and vertical structure, which in turn influence leaf trait expression. The negative association between leaf traits and species richness likely reflects management-driven functional convergence in these simplified communities. Species richness in planted forests was significantly negatively correlated with SLA, LN, and LP, indicating that in low-diversity communities, functional convergence is reinforced, and community-level traits fluctuate more strongly with the characteristics of dominant species.

The interaction between forest structure and latitude is particularly pronounced in planted forests. Latitude not only modifies the physical environment via climate but also indirectly influences stand composition and management intensity^[60]^. For example, northern planted forests are dominated by conifers adapted to cold and dry conditions, whereas southern planted forests are often composed of fast-growing broadleaf species favoring resource-acquisitive strategies^[61,62]^. This coupling of geographic context and management practices generates pronounced non-linear responses of leaf traits to latitude in planted forests. In contrast, natural forests exhibit stable community assembly through long-term niche differentiation and species turnover, resulting in markedly lower leaf trait sensitivity to latitudinal gradients.

### Multi-path interactions and integrated mechanisms

Piecewise structural equation modeling further revealed the multi-path regulatory mechanisms underlying leaf trait formation. Latitude indirectly influenced leaf traits through climate, soil, and forest structure, while its direct effects differed in direction between forest types: in natural forests, latitude exerted positive direct effects on SLA and LP, whereas in planted forests, the effects were negative (Figs 6, 7). This indicates that species turnover and functional adaptation in natural forests enhance photosynthetic investment and phosphorus utilization at higher latitudes, whereas in planted forests, constraints imposed by species composition and management practices promote more conservative resource strategies. Leaf nitrogen (LN) was primarily governed by direct stand-level effects in both forest types, with climate exerting indirect modulation, suggesting that community structure, rather than geographic position, predominantly drives nitrogen accumulation strategies. Direct fertilization records were not available for the planted forest plots in the present dataset, and large-scale forest inventory datasets seldom document such management details. Nonetheless, occasional management interventions may influence leaf nitrogen patterns; therefore, the observed latitudinal increase in LN should be viewed in light of potential management effects, as both management history and environmental gradients may jointly shape nitrogen dynamics. These findings support the 'structural mediation hypothesis', whereby latitude and climate influence functional traits through modifications of community structure^[63]^. The lack of detailed information on management history, including fertilization and rotation practices, represents a key source of uncertainty in interpreting leaf nitrogen patterns in planted forests. Therefore, the observed LN–latitude relationship should not be attributed solely to climatic or geographic gradients, as unrecorded management practices may also contribute. This underscores the need for caution in interpretation and highlights the importance of future studies integrating detailed management information.

### Limitations and future directions

At the national scale of this study, the spatial distribution of forest plots is uneven, with natural forests more common in southern China and planted forests more prevalent in northern regions, reflecting both biogeographic patterns and the legacy of large-scale afforestation programs. Accordingly, the observed latitudinal variation in leaf functional traits represents broad-scale macroecological patterns shaped by environmental gradients, together with regional land-use history and management context. In this study, forest structure was represented by three stand-level variables—stand age, density, and species richness—which capture key aspects of light competition and niche differentiation. Stand age provides a coarse indicator of developmental stage and canopy closure dynamics, influencing the spatiotemporal heterogeneity of light availability. Stand density reflects individual crowding and competitive intensity, directly linked to resource allocation, while species richness represents community compositional diversity and potential functional differentiation.

We recognize that these variables are simplified proxies of structural complexity, as they do not fully encompass vertical stratification, canopy height, basal area, or other three-dimensional attributes that critically mediate light interception and microhabitat heterogeneity. Consequently, interpretations of structure–trait relationships should be framed at a macroecological scale, acknowledging that fine-scale mechanisms may not be fully resolved. For natural forests, stand age estimates were derived from forest inventories and local forestry bureaus, a common practice in large-scale ecological analyses. Although this approach allows broad-scale inference, it introduces unavoidable uncertainty in precise age determination. Future work integrating field-measured stand age alongside multidimensional structural indices—such as canopy layering, basal area, and structural complexity metrics—will be essential to more rigorously elucidate the mechanistic links between forest structure and leaf trait expression.

Overall, the divergence in leaf trait formation mechanisms between natural and planted forests reflects two adaptive strategies: 'stability regulation' vs 'plastic response'. Natural forests achieve trait–environment optimization through long-term succession, exhibiting strong ecological resilience and functional stability. In contrast, planted forests, characterized by low species diversity and frequent anthropogenic interventions, are highly responsive to environmental variation, displaying elevated dynamic plasticity. This distinction has important implications for forest ecosystem functional stability and carbon cycling: natural forests are likely to maintain relatively stable carbon sequestration under global change, whereas planted forests may exhibit substantial functional variability in response to climatic fluctuations and management differences.

Consequently, a multivariate framework integrating climate, soil, and stand structural attributes would provide a more robust basis for interpreting how forest structure mediates leaf trait variation across broad geographic gradients. In parallel, sustained attention to nutrient constraints and soil–leaf coupling processes will be important for improving the representation of carbon, nitrogen, and phosphorus cycling in forest models. The multi-factor integrative analytical framework presented here provides new insights into the geographic regulation of forest ecosystem functions and offers valuable guidance for the sustainable management of forests in arid and temperate regions.

## Conclusions

This study reveals clear divergences in how natural and planted forests regulate leaf functional traits along latitudinal gradients, reflecting fundamentally different ecological strategies. Planted forests exhibited pronounced non-monotonic trait responses to latitude, demonstrating high environmental plasticity driven by strong sensitivity to stand structure and management-related factors. In contrast, natural forests showed only gradual latitudinal shifts in leaf traits, indicating long-term adaptive optimization shaped by climatic and edaphic filtering. These contrasting patterns arise from different underlying mechanisms. Climate and soil jointly constrained trait variation in natural forests, consistent with resource-conservation strategies and strong trait–environment coupling accumulated through succession. Planted forests, however, relied more on stand structure and exhibited decoupled structural–nutrient relationships, reflecting fast-growth strategies, shorter evolutionary history, and enhanced management effects. SEM further demonstrated that latitude influences traits primarily through indirect pathways, but the direction and strength of these pathways differ sharply between forest types, underscoring divergent ecological controls on leaf economics. Theoretically, the present findings highlight that forest origin alters the balance between environmental filtering and plastic adjustment, shaping macroecological trait patterns across biomes. Practically, the high sensitivity of planted forests traits to structural and climatic variability suggests that enhancing stand complexity, species diversity, and soil nutrient regulation is essential to improve the functional stability and carbon sequestration capacity of planted forests under ongoing global change. The present findings provide actionable insights for climate-adaptive plantation design. Specifically, tree species selection and planting density can be optimized by prioritizing trait combinations that balance resource acquisition and conservation, and implementing site-specific management and regeneration strategies to enhance planted forests' stability and productivity under future warming and variable moisture conditions.

## SUPPLEMENTARY DATA

Supplementary data to this article can be found online.

## Data Availability

The datasets generated during and/or analyzed during the current study are available from the corresponding author upon reasonable request.
